# Cellular stress response to extremely low‐frequency electromagnetic fields (ELF‐EMF): An explanation for controversial effects of ELF‐EMF on apoptosis

**DOI:** 10.1111/cpr.13154

**Published:** 2021-11-06

**Authors:** Mojdeh Barati, Behrad Darvishi, Mohammad Amin Javidi, Ali Mohammadian, Seyed Peyman Shariatpanahi, Mohammad Reza Eisavand, Alireza Madjid Ansari

**Affiliations:** ^1^ Integrative Oncology Department Breast Cancer Research Center Motamed Cancer Institute ACECR Tehran Iran; ^2^ Recombinant Proteins Department Breast Cancer Research Center Motamed Cancer Institute ACECR Tehran Iran; ^3^ Department of Medical Biotechnology Faculty of Medical Sciences Tarbiat Modares University Tehran Iran; ^4^ Institute of Biochemistry and Biophysics University of Tehran Tehran Iran

**Keywords:** apoptosis, cellular stress response, controversy, ELF‐EMF, reactive oxygen species

## Abstract

Impaired apoptosis is one of the hallmarks of cancer, and almost all of the non‐surgical approaches of eradicating tumour cells somehow promote induction of apoptosis. Indeed, numerous studies have stated that non‐ionizing non‐thermal extremely low‐frequency magnetic fields (ELF‐MF) can modulate the induction of apoptosis in exposed cells; however, much controversy exists in observations. When cells are exposed to ELF‐EMF alone, very low or no statistically significant changes in apoptosis are observed. Contrarily, exposure to ELF‐EMF in the presence of a co‐stressor, including a chemotherapeutic agent or ionizing radiation, can either potentiate or inhibit apoptotic effects of the co‐stressor. In our idea, the main point neglected in interpreting these discrepancies is “the cellular stress responses” of cells following ELF‐EMF exposure and its interplay with apoptosis. The main purpose of the current review was to outline the triangle of ELF‐EMF, the cellular stress response of cells and apoptosis and to interpret and unify discrepancies in results based on it. Therefore, initially, we will describe studies performed on identifying the effect of ELF‐EMF on induction/inhibition of apoptosis and enumerate proposed pathways through which ELF‐EMF exposure may affect apoptosis; then, we will explain cellular stress response and cues for its induction in response to ELF‐EMF exposure; and finally, we will explain why such controversies have been observed by different investigators.

## Graphical abstract

The outcome of cancer cells exposure to ELF‐EMF, as cellular stress response, is closely related to ER stress, NF‐κB inflammatory, Heat Shock Protein, Sirtuin, and DNA repair responses. The controversial evidence of ELF‐EMF‐induced apoptosis or autophagy in cancer cells has been hypothetically described by the way these cellular feedback happen.

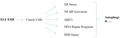

## 1 INTRODUCTION

In modern world, electromagnetic fields (EMFs) have become an inseparable part of routine life. Numerous electric power‐generating human‐made devices are now producing EMFs which are overlaid on those of earth's magnetic field. EMFs are usually identified with a 50 or 60 Hz frequency and therefore are classified under the extremely low‐frequency, non‐ionizing span of electromagnetic spectrum.^1^ Due to these physical characteristics, ELF‐EMFs are not capable of breaking molecular bond or inducing thermal effects on tissue. However, it is now proven that they can interact with human tissues and induce some weak electrical currents.^2^ In addition, it is not completely understood whether biological effects induced by EMFs are hazardous for human or environment. During last few decades, a number of studies have reported beneficial effects of ELF‐EMFs in treatment of cancer both in vitro and in vivo.^3^, ^4^, ^5^, ^6^, ^7^ Despite this, the exact mechanism of these anti‐neoplastic effects has not been confirmed yet.

So far, the most probable mechanism proposed for explaining anticancer effects of ELF‐EMF is induction of apoptosis through upregulation of intracellular reactive oxygen species (ROS) which has also been confirmed by different experimental studies. In the study performed by Ding et al.,^8^ it was demonstrated that 24‐h exposure to 60 Hz, 5 mT ELF‐EMF could potentiate apoptosis induced by H_2_O_2_ in HL‐60 leukaemia cell lines. Similarly, in the study performed by Jian et al.,^9^ exposure to an intermittent 100 Hz, 0.7 mT EMF significantly enhanced rate of apoptosis in human hepatoma cell lines pretreated with low‐dose X‐ray radiation. Kaszuba‐Zwoinska et al.^10^ also showed that short‐term exposure of human acute monocytic leukaemia cell line exposure to 50 Hz, 45 ± 5 mT pulsed EMF, significantly potentiated rate of apoptosis induced by cyclophosphamide and colchicine. Benassi et al. reported that co‐treatment of human ovarian adenocarcinoma cell lines with cisplatin, a chemotherapeutic agent with DNA‐damaging and ROS‐promoting activity, significantly enhanced sensitivity to apoptosis through increasing both caspases 3 and 9 activity. This is in accordance with previous studies demonstrating an enhancement in 1‐methyl‐4‐phenylpyridinium (MPP +) induced caspase‐dependent apoptosis following 24‐h exposure to 50 Hz, 1 mT ELF‐MF in SH‐SY5Y neuroblastoma cell lines.^11^

One of the main mechanisms proposed for defining anticancer effects of ELF‐EMF is induction of apoptosis through upregulation of reactive oxygen species (ROS) which has also been confirmed by different experimental studies.

Contrary to above‐mentioned studies, several reports propose an anti‐apoptotic activity for ELF‐EMF. Pirozzoli et al.^12^ reported that 24‐h exposure to 50 Hz, 1 mT ELF field significantly attenuated apoptosis induced by camptothecin in LAN‐5 neuroblastoma cell lines. De Nicola et al reported that puromycin‐induced apoptosis in human lymphoblasts was significantly weakened in response to 2‐h exposure to a 0.1 mT ELF field.^13^ They reported that reduced glutathione (GSH) was the key mediator of the observed effect. In addition, based on the study performed by Palumbo et al.,^14^ pretreatment of Jurkat leukaemic cell lines with 50 Hz, 1 mT EMF resulted in 22% reduction in caspase 3‐dependent apoptosis induced by anti‐Fas therapy. Moreover, based on Cid et al.,^15^ the anti‐apoptotic activity of melatonin on HepG2 cell lines was completely abrogated in response to 42‐h intermittent exposure with a 50 Hz, 10 µT EMF. Similarly, bleomycin‐induced apoptotic activity in K562 erythroleukaemia cell line was significantly reduced in response to a short‐term (~10 min) exposure period to a 217 Hz, 120 µT ELF‐MF.^16^ Based on Brisdelli et al.,^17^ concurrent treatment of K562 cell lines with ELF‐EMF and quercetin for 24 h significantly increased expression of Bcl2, a protein with anti‐apoptotic activity, compared with quercetin alone treated and control groups. They also reported that extending ELF‐EMF exposure for 1–3 days results in attenuation of growth inhibitory effects of quercetin in leukaemia cell lines which was in association with reduced level of caspase 3 activity, along with inhibition of quercetin induced reduction in expression of Bcl‐xL and Mcl‐1 anti‐apoptotic proteins.

Still, some reports have stated no statistically significant cytotoxic or cytostatic activity for ELF‐EMF. Laqué‐Rupérez et al.^18^ reported no statistically significant changes in methotrexate‐induced cytotoxicity in MCF‐7 breast cancer cell lines after exposing them to 25 Hz, 1.5 mT pulsed EMF. Similarly, in the study performed by Mizuno et al.,^19^ no statistically significant changes in survival rates of SV40 cells were observed between cells which were subjected to UV radiation alone and group subjected to concurrent administration of 24‐h 60 Hz, 5 mT EMF and UV radiation. Finally, Höytö et al.^20^ reported no statistically significant enhancement in anti‐proliferative and cytotoxic activities of menadione on SH‐SY5Y neuroblastoma cells when combined with 24 h exposure to ELF‐MF of 100 µT intensity.

This discrepancy in observations has made it difficult to come into a unit conclusion, and therefore, application of ELF‐EMF in clinic for treatment of cancer still remains a big dilemma. In our idea, the main point neglected in interpreting the discrepancies observed in results is consideration of cellular stress responses induced by ELF‐EMF exposure and its interplay with the molecular mechanisms underlying apoptosis. The main purpose of current review was to outline the triangle of ELF‐EMF, cellular stress response of cells and apoptosis, and interpret and unify the discrepancies in results based on this theory. Therefore, initially we will explain studies performed on identifying the effect of ELF‐EMF on induction/inhibition of apoptosis, enumerate proposed pathways through which ELF‐EMF exposure may affect apoptosis; then, we will explain cellular stress response, cues for activation of this phenomenon in response to ELF‐EMF exposure and finally under a separate “discussion” section we will try to explain why such controversy has been obtained by different investigators.

## 2 APOPTOSIS AND ELF‐EMF EXPOSURE

Considering hallmarks of cancer, aberrant cellular survival is an important characteristic of malignant cells which is usually attributed to a mis‐regulated apoptotic state in cells. Apoptosis is a type of programmed cell death which is abundantly observed under both physiological and pathological conditions, upon interaction of cells with specific stimulators, capable of activating either of intrinsic and extrinsic pathways. Moreover, failure in induction of apoptosis, as a consequence of aberrant expression of antigens, secreted angiogenic growth factors, or their receptors has already been linked to an elevated risk of metastasis, promotion of angiogenesis and an accelerated risk of resistance development to anti‐angiogenic cancer therapies.^21^, ^22^, ^23^, ^24^, ^25^, ^26^, ^27^ Either mediated by the extrinsic (mediated by FASL, TNFα and so on) or intrinsic pathway (most importantly, accumulation of ROS and development of oxidative stress), the rest of the process will be followed by modulation of specific sets of procaspase molecules cleavage (caspase 8 and caspase 9 for extrinsic and intrinsic pathways respectively), ending in degradation of numerous intracellular target proteins, blebbing of cellular membrane, cleavage and degradation of chromosomal DNA, and finally, getting phagocytosed and scavenged by polymorphonuclear cells.^28^ Apoptosis can be triggered upon activation of two main pathways which are broadly referred as “intrinsic” and “extrinsic” pathways.

The most prevalent mechanism through which several chemotherapeutic agents trigger apoptosis is induction of mitochondrial membrane permeabilization, the intrinsic apoptosis pathway, which is mainly controlled by Bcl‐2 proteins family. This process results in leakage of several pro‐apoptotic molecules such as cytochrome *c*, Smac/DIABLO, apoptosis‐inducing factor (AIF) and endonuclease G (Endo G) into the cytoplasm.^29^ Released Endo G and AIF initiate nuclear modifications while the others activate caspases. Cytochrome *c* promotes formation of apoptosis protease activating factor‐1 (Apaf‐1) oligomers using ATP or dATP.^30^, ^31^ This complex in next place recruits procaspase 9 and forms “apoptosome” which in turn induces autoactivation of procaspase 9.^32^, ^33^ Matured caspase 9 further activates caspase 3 and 7 which in turn results in initiation of downstream caspase cascades^34^ and induction of apoptotic cell death. In parallel, Smac/DIABLO antagonize suppressing effects of inhibitors of apoptosis proteins (IAPs) on activated caspases.^35^, ^36^

In some cell types however, chemotherapeutic‐induced apoptotic cell death may be initiated through the death receptor Fas (APO‐1/CD95), the extrinsic apoptosis pathway. Ligation of Fas with its natural ligand, FasL, promotes Fas clustering, which in next place attracts FADD^37^ and procaspase 8,^38^ totally forming a complex referred as death‐inducing signalling complex (DISC). The mature caspase 8 would be exhausted from the DISC after oligomerization and​autoactivation of procaspase 8.^39^ Based on the cell type, mature caspase 8 initiates apoptosis by two distinct pathways.^40^ In first pathway, high quantities of mature caspase 8 induce direct cleavage and activation of procaspase 3 without enrolment of mitochondrial pathway. In second pathway however, low quantities of mature caspase 8 are formed which is not capable of directly inducing activation of procaspase 3. Alternatively, herein, caspase 8 promotes cleavage of the “BH3‐only protein” Bid and formation of truncated Bid which, in turn, triggers mitochondrial apoptosis pathway.^41^, ^42^

Different groups of anticancer agents are capable of activating death receptor pathway through enhancement of Fas or FasL expression.^43^ This process is transcription‐dependent and involves p53 activity.^44^ The activated signalling pathway following Fas/FasL complexation outlines an autocrine/paracrine pathway like that happening during activation‐induced cell death in T lymphocytes. Nevertheless, FasL plays minimal role in chemotherapy‐induced apoptosis, as administration of antagonist antibodies or any small molecule preventing from FasL/Fas interaction does not suppress apoptosis.^45^ Likewise, the pro‐apoptotic effects of chemotherapeutic agents on embryonic fibroblasts from FADD and caspase 8 knockout mice remained unaltered.^46^, ^47^

Although apoptosis is usually induced upon overproduction of ROS and development of oxidative stress, a mild‐to‐moderate level of ROS is required for maintenance and regulation of physiological function of cells including growth, proliferation, differentiation and migration^52^; regulation of immune system's function and maintaining redox balance^48^; and promotion of autophagy through activation of different signalling pathways including phosphoinositide 3‐kinase (PI3K)/Akt, mitogen‐activated protein kinases (MAPK), nuclear factor (erythroid‐derived 2)‐like 2 (Nrf2)/Kelch‐like ECH‐associated protein 1 (Keap1), nuclear factor‐κB (NF‐κB) and the tumour suppressor p53.^48^, ^49^, ^50^, ^51^ Hence, manipulation of ROS level in cells is a good strategy for cancer therapy.

If ROS generation and accumulation can be considered the first cellular event of ELF‐EMFs exposure, the modification of intracellular Ca^2+^ levels could be one of the most important mechanisms by which ROS have their multiple actions in cells.^52^ Over the past few years, lots of data have shown that ELF‐EMF exposure regulates intracellular Ca^2+^ level which can, in turn, activate multiple physiological mechanisms such as differentiation of chromaffin cells into neuronal‐like cells (ELF‐MF, 60 Hz, 0.7 mT, 2 h/day twice a day^53^); cell death by apoptosis (ELF‐MF, 50/60 Hz, 0.2–5 mT, 2–3 consecutive days^54^, ^55^); functional modification of the immune system's cells through involvement of P2Y membrane receptors (sinusoidal electric fields, 0.3 or 30 kV/m, 50 Hz, for 24 h^56^), activation of mechanically operated stretch‐activated Ca^2+^ channels (noninvasive electrical stimulus, 0.1‐V/cm direct current^57^); and the enhancement of the expression of voltage‐gated Ca^2+^ channels in different human cell systems (static magnetic fields, 0.15 and 66 mT^58^). In this context, Kapri‐Pardes et al. examined responses of cells (both transformed and non‐transformed) to ELF‐EMFs across a broad range of field strengths by examining activation of ERK1/2 and other signalling pathways. They reported that all cell lines could sense and respond to ELF‐EMFs. Nevertheless, the extent to which transformed cells responded to EMFs was significantly lower compared to non‐transformed ones, and interestingly, in MDA‐MB‐231 cells, exposure decreased phosphorylation of ERK1/2. Perhaps the more important finding of their study was that contrary to what previously was though, cells can sense magnetic field strengths as low as 0.15 µT which is at least partly mediated through activation of NADH oxidase.^59^

So far, multiple signalling pathways have identified to be affected under acute or short‐term exposure to ELF‐EMF. Indeed, exposure to ELF‐EMF promotes tyrosine phosphorylation of specific protein components of signalling pathways in cells. For instance, it has been shown that 1‐ to 30‐min exposure to 60 Hz, 0.1 mT ELF‐EMFs results in activation of Lyn, a protein tyrosine kinase and serine/threonine kinase protein kinase C (PKC) in B lymphocytes.^60^ Likewise, acute exposure of Jurkat cell line (~5 min) to 50 Hz, 0.1 mT ELF‐EMFs activates Lck, which in turn promotes complexation of T cell receptors.^61^ Similar result was also reported in adherent cells, where 5 min exposure to 50 Hz, 04 mT ELF‐EMFs promoted epidermal growth factor receptor (EGFR) clustering and subsequent stimulation of Ras GTPases in long fibroblast cells of Chinese hamster.^62^ In addition, cyclic AMP/protein kinase A (cAMP/PKA) is another pathway which is activated in response to exposure to ELF‐EMF in rat's cerebellar granule cells and human skin fibroblasts.^63^, ^64^

Mitogen‐activated protein kinase (MAPK) cascades are among the other important signalling cascades which are stimulated upon exposure to ELF‐EMF in several types of examined cells.^65^ MAPK pathways consisting from four main cascades, including extracellular signal regulated kinase 1 and 2 (ERK1/2), ERK5, p38 and c‐Jun N‐terminal kinase (JNK), are central in regulation of almost all stimulated cellular events such as differentiation, proliferation, stress responses and apoptosis.^65^, ^66^ After initial stimulation, these cascades function by serially activating specific protein kinases in each level of cascade with ultimate result of phosphorylation of thousands of target proteins and modulation of related cellular processes. Akt is another protein kinase with responsibilities similar to the MAPKs.^67^ Akt becomes activated in response to extracellular stimuli upon phosphorylation of its two activatory moieties following interaction with PI3K‐phosphorylated phospholipids. Any dysregulation or abnormalities in mentioned five signalling pathways is associated with certain disorders including cancer.^67^, ^68^ Interestingly, both acute/short‐term and chronic/long‐term exposure to ELF‐EMF has shown to induce activation of Akt and MAPK.^69^, ^70^ For instance, 30‐min exposure to ELF‐EMF results in activation of ERKs and Akt in several cancer cell lines including MCF7, HaCaT, NB69, HL‐60 and so on.^69^, ^70^, ^71^, ^72^ Furthermore, 3‐ to 15‐min exposure to 50 Hz in CHL cells and 15, 30, or 60 min in NB69 cells results in activation of stress‐associated MAPKs, p38 and JNK.^70^, ^73^, ^74^

In most of these studies, the strengths of applied ELF‐EMFs were more than 100 µT, and none has investigated the relation between the changes in strength of EMFs and induction of cell signalling cascades. Recently, Kapri‐Pardes et al. examined responses of cells (both transformed and non‐transformed) to ELF‐EMFs across a broad range of field strengths by examining activation of ERK1/2 and other signalling pathways. They reported that all cell lines could sense and respond to ELF‐EMFs. However, the extent to which transformed cells responded to EMFs was significantly lower compared to non‐transformed ones, and interestingly, in MDA‐MB‐231 cells, exposure decreased phosphorylation of ERK1/2. Perhaps the more important finding of their study was that contrary to what previously was though, cells can sense magnetic field strengths as low as 0.15 µT which is at least partly mediated through activation of NADH oxidase.^59^

Although effects of EMF exposure on TGF‐β/BMP signalling pathway have been studied during the process of bone repair, same pathway is a key player in pathophysiology of cancer and its modulators demonstrate statistically significant anti‐metastatic activities.^75^ Different studies have shown that exposure to pulsed EMF results in a statistically significant increase in TGF‐β, in osteoblastic cells and both atrophic and non‐hypertrophic cells.^76^, ^77^ In addition, based on a recent study, exposing differentiating osteoblasts to pulsed EMF, promotes activation of TGF‐β signalling pathway through Smad2 and increases expression of osteoblastic differentiation markers such as ALP and type I collagen.^78^ BMP expression during osteogenesis was also increased after exposure to pulsed EMFs.^79^, ^80^, ^81^ Moreover, it has been shown that exposure to pulsed EMFs, stimulates osteogenic differentiation and maturation through the activation of BMP‐Smad1/5/8 signalling. In this case, BMP receptor II, BMPRII, regulates differentiation in a cilium‐dependent manner.^82^ Considering the separate effects of BMP and pulsed EMFs on differentiation and maturity of osteoblasts, many studies have shown that concurrent treatment with BMP and pulsed EMF enhances bone formation to a much greater degree compared to each treatment alone.^83^, ^84^, ^85^, ^86^

In addition to the mentioned signalling pathways, electromagnetic fields can also affect pathways underlying VEGF and FGF signalling molecules.^87^, ^88^ Based on a recent report, exposure to pulsed EMF significantly increases expression of IGF‐1 at mRNA level and promotes bone formation.^89^ In addition, pulsed EMF (1.5 mT, 75 Hz) can also increase synthesis of proteoglycans and protect human articular cartilage from further damage.^90^ Finally, it has been shown that exposure to pulsed EMF reverses osteoporotic effect of dexamethasone.^91^

Notch signalling is a highly conserved pathway that regulates cellular fate and skeletal development. Recent reports have shown that exposure to pulsed EMF can regulate expression levels of Notch4 receptor, as well as DLL4 ligands and target genes (Hey1, Hes1 and Hes5) during the osteogenic differentiation of human mesenchymal stem cells. Interestingly, expression of osteogenic markers, including Runx2, Dlx5, Osterix, Hes1 and Hes5, after pulsed EMF treatment was reversed following treatment of cells with notch pathway inhibitors.^92^ Furthermore, exposure to pulsed EMF significantly increases the level of cAMP, protein kinase A activity and accelerates osteogenic differentiation of MSCs.^93^, ^94^ Anti‐inflammatory effects of pulsed EMFs have also been reported both in vitro^95^, ^96^ and in vivo,^97^, ^98^, ^99^ as well as in clinical settings.^100^

## 3 ELF‐EMF AND INDUCTION OF CELLULAR STRESS RESPONSE

Numerous studies have shown that cells are physiologically well buffered against negative effects of ELF‐EMF alone. However, in the presence of stressful condition, including exposure to toxins, viruses, DNA damage and proteotoxic, hypoxic, metabolic and oxidative stress, an additional weak stressor like ELF‐EMF might produce large effects.^101^ Based on Mattsson and Simko who extensively investigated the oxidative response of cells following ELF‐EMF exposure, ROS levels can be consistently altered in different cell types or experimental conditions following exposure to magnetic fields. These effects were prominent for fields with intensities more than 1 mT, but were also documented at or below 100 mT. Despite this, all observed effects where moderate and majority of changes were below 50%.^102^ Consequently, the produced amounts of ROS by ELF‐EMF are not high enough to induce major DNA damage. Although this mild elevation in ROS levels in response to acute or chronic exposure to ELF‐EMF cannot trigger cell death, it may induce cellular resistance against oxidative damage through upregulation of antioxidant pathways and induction of cellular stress response. Small change in ROS levels stated above is capable of promoting different cell signalling pathways especially by means of superoxide ions.^20^, ^103^, ^104^, ^105^, ^106^ This phenomenon requires a certain time to develop and promotes several other time‐dependent changes.^105^

As discussed earlier, antioxidant defence capacity of cells can be changed following exposure to ELF‐EMF. For example, it has been shown that exposure to ELF‐EMF can significantly increase SOD levels in cells.^107^ Furthermore, ELF‐EMF can enhance activity of both glutathione‐S‐transferase and ‐reductase enzymes in malignant cells.^108^ Also, based on Cichon et al.,^109^ ELF‐EMF exposure can upregulate expression of different antioxidant target genes including CAT, SOD1, SOD2, GPx1 and GPx4. In addition, multiple pathways involved in orchestrating cellular stress response of cells to stressful condition can also become activated following ELF‐EMF exposure. Based on literature, generation of mitochondrial ROS at the time of ELF‐EMF exposure is pivotal for activation of signalling pathways involved in cellular adaption.^110^ Activation and upregulation of Nrf2 expression, the master redox‐sensing transcription factor may be the most prominent example in this regard which has been confirmed in a Huntington's disease‐like rat model.^111^ Another cellular stress response to ELF‐EMF involves activation of MAPK and NF‐κB which, in turn, may upregulate expression of peroxisome proliferator‐activated receptor‐γ coactivator‐1α (PGC‐1α) and enhance mitochondrial biogenesis.^112^ Activation of autophagy, ER stress, heat‐shock response and sirtuin 3 expression are among the other identified cellular stress responses to ELF‐EMF exposure, all of which have been discussed earlier.

This cellular stress response is very important when ELF‐EMF exposure is applied before chemotherapy. The main mechanism through which several chemotherapeutic agents induce apoptosis in cancer cells is elevation of ROS and induction of apoptosis. However, as antioxidant defence after ELF‐EMF exposure is enhanced, cells become more resistant to these agents. Such effects have also been reported during radiation therapy and are responsible for development of resistance to radiotherapy. Contrarily, when chemotherapy and ELF‐EMF exposure are performed simultaneously, this increase in ROS levels potentiates the oxidative stress induced by chemotherapeutic agents, as the ROS levels become excessively high and cells do not have time for adaption. Therefore, the result is enhancement of apoptosis. Differences between extent of apoptosis induced or when no significant differences are observed in combination are mostly dependent on the nature of the cell (ie the antioxidant defence), type and dose of the chemotherapeutic agent applied and the number of cells seeded in the plate.

A number of other harmful agents or conditions, such as thermal stress,^113^ exposure to alkylating agents,^114^ heavy metals^115^ and ionizing radiation^116^ have shown to initiate a similar response. Generally, cellular stress response is characterized by modulation of expression of various genes. The main outcome of this alteration in pattern of gene expression is protection of cells from cytotoxic doses of a harmful agent. This response represents that following exposure to a toxin, cells expect or at least prepare themselves for a lethal concentration of the agent. In addition to mild exposure to toxic agents or stressful conditions, physiological conditions may also promote development of an cellular stress response.^117^ For instance, exercise training reduces the extension of lipid peroxidation during acute exercise which has been attributed to induction of oxidative stress.^118^, ^119^ Likewise, an enhanced repairing capacity was observed in lymphocytes of workers which were occupationally become exposed with low levels of ionizing radiation.^120^

It is now clear that sub‐lethal doses of oxidants are capable of inducing cellular stress responses in cells. This phenomenon was initially discovered in bacteria, but now it has also been documented in eukaryotic cells.^121^, ^122^, ^123^, ^124^ The protective responses induced in cells during challenge with sub‐lethal doses of oxidants have been identified in three major systems. These include hydrogen peroxide (H_2_O_2_) and superoxide anion (O2^−^)‐induced reactions in bacteria, protective responses induced by sub‐lethal doses of oxidants in eukaryotic cells which render them resistant to lethal doses of the same or a related oxidant and finally protective responses induced by sub‐lethal doses of oxidants in eukaryotic cells which render them resistant to lethal doses of other toxic agents. Overall, cells possess two primary defence mechanisms against oxidative stress. The first includes cellular molecules or enzymes that directly participate in scavenging free radicals and preventing oxidative stress‐induced damage to cells such as catalase, superoxide dismutases (SOD), glutathione peroxidases, ascorbate and glutathione. The second line, however, consists of enzymes involved in repairing or scavenging oxidatively damaged macromolecules such as DNA and proteins. Typical examples of such enzymes are DNA nucleases and glycosylases.^117^

## 4 MECHANISMS UNDERLYING ELF‐EMF‐MEDIATED CELLULAR STRESS RESPONSE

**FIGURE 1:**
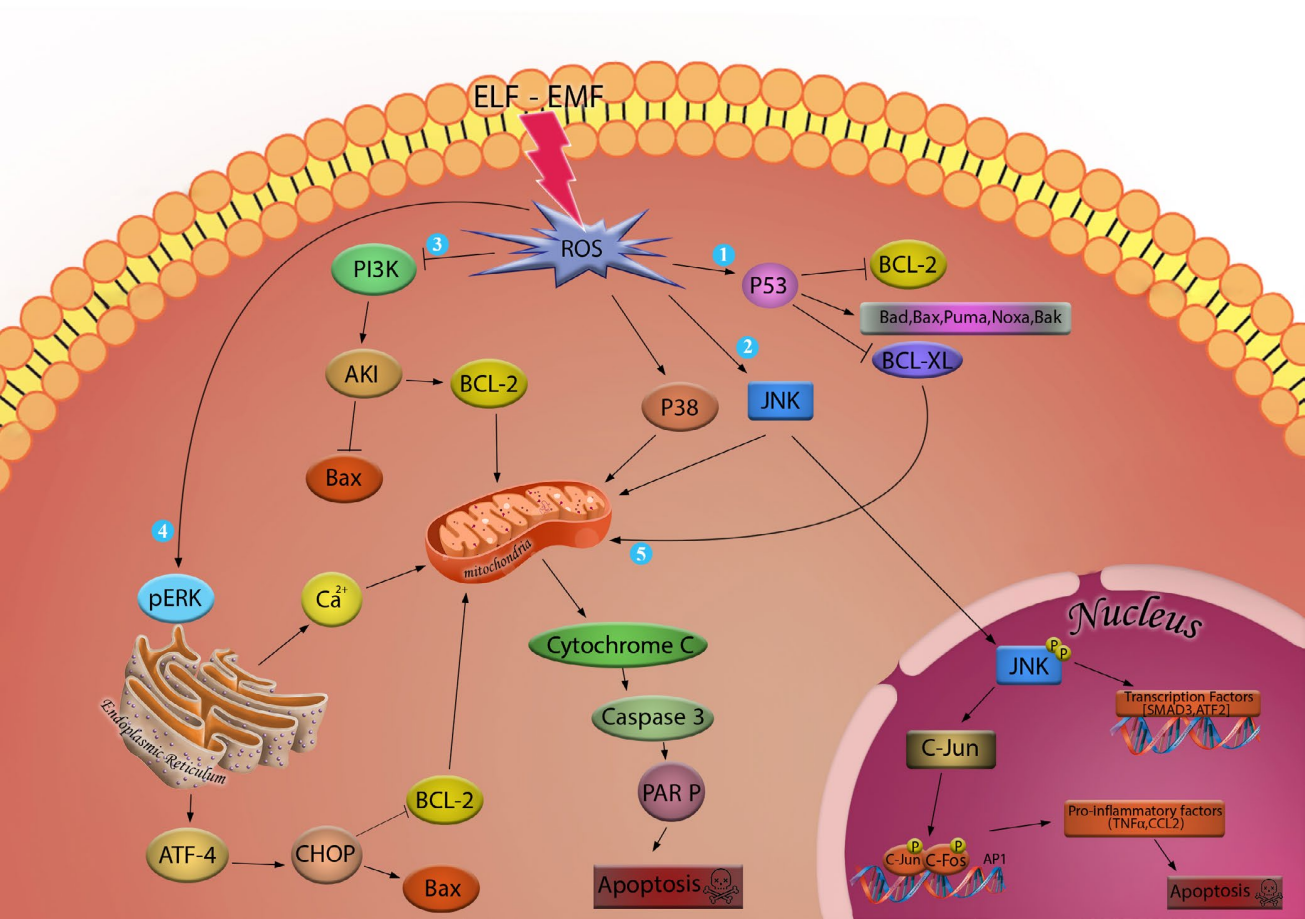
ROS‐mediated apoptosis signalling pathways: (1) Accumulation of ROS affects p53 protein which in turn inhibits Bcl‐2 and Bcl‐XL proteins function and promotes the activity of Bad, Bax, Bak, Puma and Noxa proteins. (2) ROS can induce phosphorylation of JNK. Phosphorylated JNK can activate transcription factors such as SMAD3 and ATF2. Phosphorylated JNK can also translocate to the nucleus and activate C‐Jun phosphorylation which in turn can activate transcription of several pro‐apoptotic factors. (3) Accumulation of ROS inhibits PI3K‐mediated activation of AKT. (4) Accumulated ROS promotes ER stress and expression of CHOP through activation of ATF‐4 which in turn can promote Bax activity and inhibit Bcl‐2. (5) All these pathways end in the release of cytochrome c which in turn can activate caspase 9 and caspase 3 and result in cleavage of PARP and induction of apoptosis

**FIGURE 2:**
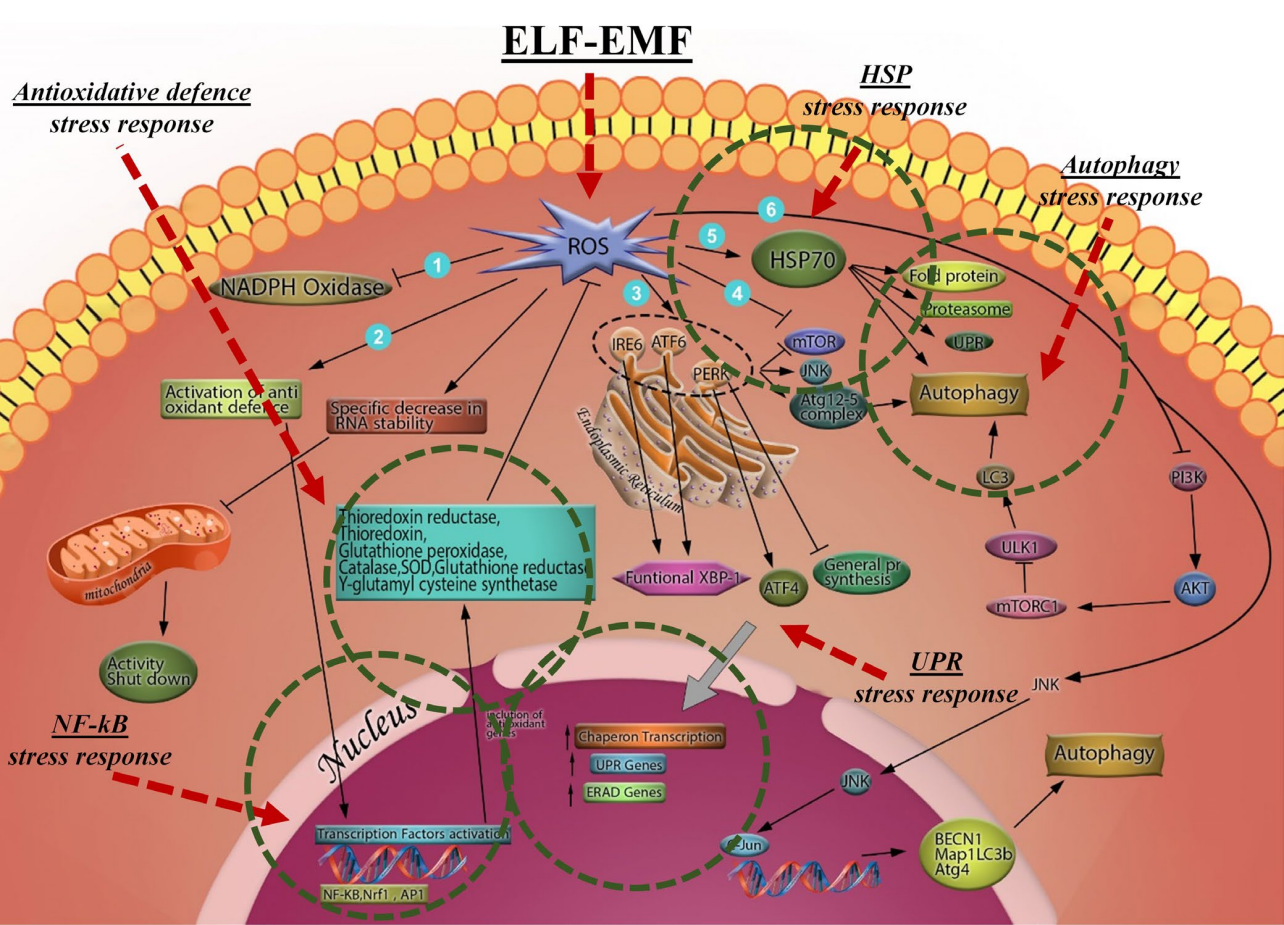
ROS‐mediated cellular stress response: (1) mild accumulation of ROS inhibits NADPH oxidase activity. (2) Mild accumulation of ROS activates antioxidant defence system which involves activation of transcription factors including NF‐kB, Nrf‐1 and AP‐1 which in turn upregulates expression of thioredoxin reductase, glutathione peroxidase, SOD, etc., which can suppress further accumulation of ROS. (3) Mild accumulation of ROS activates ER stress through affecting IRE6, ATF6 and PERK. PERK in turn inhibits general protein synthesis and ATF4 and functional XBP‐1 promote chaperon transcription, UPR genes and ERAD genes which can protect cells against accumulated ROS. (4) Mild accumulation of ROS can directly induce autophagy through inhibition of mTORC. (5) Mild accumulation of ROS can upregulate expression of HSP70 which can affect protein folding, proteasome activation and induction of autophagy. (6) Mild accumulation of ROS can also activate JNK and after that c‐JUN which can in turn activate BECN1, Atg4 and MAP1LC3B genes expressions, most important proteins involved in autophagy. ROS can also inhibit PI3K pathway and modulate autophagy. Finally, mild accumulation of ROS can induce specific decrease in RNA stability and result in mitochondrial activity shut down

The cellular stress response to oxidative stress in mammalian cells consists of seven main pathways including unfolded protein response (UPR), antioxidant response, heat‐shock response, autophagic response, NF‐kB inflammatory response, sirtuin response and DNA repair response. Numerous studies in literature have reported that exposure to ELF‐EMF can activate most of these pathways without inducing significant increase in cell death or apoptosis both in normal and in cancer cells (Table 1). Here, we will comprehensively review the ways through which cells respond to elevated ROS following exposure to ELF‐EMF and orchestrate cellular stress response (Figures 1 and 2).

**TABLE 1:** Different cellular stress responses affected by ELF‐EMF exposure

Experiment performed	ELF‐EMF treatment	Cell line	Observed effects
1. Heat‐shock protein response			
Corallo et al.^140^	100 Hz	Primary osteoarthritic chondrocytes	Increased Mn‐superoxide‐dismutase and heat‐shock proteins expression
Alfieri et al.^141^	50 Hz, 0.68 mT	Endothelial cells	A poor and transient activation of HSF1
Frahm et al.^142^	50 Hz, 1 mT	Mouse macrophages	Hsp70 and Hsp110 exhibited increased levels at certain time point
Wei et al.^143^	15 Hz, 2 mT	Hypoxic cardiomyocytes	Significantly increased HSP70 mRNA expression
Bernardini^144^	50 Hz	Porcine aortic endothelial cells	Increase in the mRNA levels of HSP70 No increase in Hsp27, Hsp70 and Hsp90 protein levels
Akan et al.^145^	50 Hz, 1 mT	THP‐1 cells	Increased hsp70 levels in a time‐dependent manner
2. Unfold protein response			
Chen et al.^161^	Picosecond pulsed electric fields	HeLa cells	Affected the phosphorylation levels of endoplasmic reticulum sensors and upregulated the expression of GRP78, GRP94 and CHOP
Keczan et al.^162^	PEMF	HEK263T	No remarkable effect
		HepG2	No remarkable effect
		HeLa	Increased BiP, Grp94 and CHOP expression
3. Autophagy			
Chen et al.^173^	Pulsed electromagnetic fields (2 mT, 50 Hz)	Embryonic fibroblasts (MEF)	A significant increase in autophagic biomarkers including LC3‐II and formation of GFP‐LC3 puncta was observed
4. NF‐kB activation			
Kim et al.^204^		RAW264.7 cells	Enhanced translocation of phosphorylated NF‐κB in to the nucleus and induction of inflammatory responses
5. SIRT3 activation			
Falone et al.^196^	ELF‐EMF 1 mT, 50 Hz	SH‐SY5Y	Upregulation of the major sirtuins, increased signalling activity of the NRF2

### 4.1 Heat‐shock response

In most eukaryotes, heat‐shock factors (HSF) [ie transcription factors that regulate expression of heat‐shock proteins (HSPs)] are located in cytoplasm in bond with HSP70, HSP90 or other proteins which renders them to be inactive during normal condition.^125^, ^126^ During stressful condition however, cells are exposed to a much higher extent of denatured proteins. In this condition, as HSPs prefer to act more like a molecular chaperone instead of a regulatory protein, they become detached from HSF and undergo oligomerization. In next step, oligomerized HSFs translocate to the nucleus where they promote expression of *HSP* and related heat‐responsive genes.^127^, ^128^ Different studies have shown that treatment of cells with H_2_O_2_ and induced ROS can increase expression of heat‐responsive genes.^129^, ^130^, ^131^ In the study performed by Volkov et al.^128^ for example, it was shown that heat treatment and H_2_O_2_ elevate expression of AtHSP17.6 and AtHSP18.6 genes up to a similar level. In this regard, it has been hypothesized that heat may also activate HSFs by elevation of ROS in an indirect manner. Consistent with this finding, it has been shown that sub‐lethal amounts of ROS induced by thermal stress can enhance segregation of HSP‐HSF complexes.^126^ In addition, certain HSFs have shown to play as a sensor for H_2_O_2_.^132^, ^133^

Among different ROS, H_2_O_2_ is the main player in modulating signalling pathways partly owing to its moderate reactivity and consequently long half‐life and stability.^134^ Furthermore, produced H_2_O_2_ can also easily pass through membrane and therefore take role of a signalling molecule.^135^ Based on Miller and Mittler, H_2_O_2_ may also trigger HSF’s trimerization through direct modification of HSFs. In addition, MAPK is another pathway through which ROS and HSFs may communicate with each other since HSF phosphorylation has been identified both in mammals and in yeasts.^136^, ^137^, ^138^ Finally, oxidative stress is capable of promoting assembling and formation of high molecular weight HSE‐binding complexes which are the hallmarks of early HSFA1a/A1b‐dependent gene expression in thermal stressed leaves of Arabidopsis.^128^, ^139^

Exposing several primary or primary‐like cell lines to ELF‐EMF has shown to change HSP levels. These effects were identified both by upregulation of *HSP* genes mRNA levels and protein amounts in different cell types including human chondrocytes,^140^ fibroblasts (HuDe, WI‐38)^141^ and endothelial cells (SPAE, HUVECs), as well as mouse macrophages,^142^ rat neonatal cardiomyocyte^143^ and porcine aortic endothelial cells.^144^ HSP expression was also induced following exposure of several human lymphoma and leukaemia cell lines including K562, HL‐60, U937, CEM and THP‐1 to ELF‐EMF.^106^, ^141^, ^145^, ^146^, ^147^ As the energy required for partially unfolding of a protein is about 14 orders of magnitude higher than those possessed by magnetic fields energy, it is very unlikely that ELF‐EMF exposure cause (partial) unfolding in proteins.^101^ Therefore, indirect pathways including promotion of ROS accumulation may be the main pathway through which ELF‐EMFs may affect folding of proteins and induce cellular stress responses.

### 4.2 Unfold protein response

In order to function properly, proteins require a specific three‐dimensional folding.^148^, ^149^ This unique structural folding is mainly stabilized through intramolecular disulphide bonds particularly for membranous and secretory proteins.^150^ Endoplasmic reticulum (ER) is the specific place where nascent proteins synthesized by cytoplasmic ribosomes translocate and become folded and functional.^151^ The main by‐product of the process of protein folding is H_2_O_2_ which results in maintenance of a high level of ROS.^152^, ^153^ Therefore, ER redox state is in close relation with correct ER functioning and maintenance of ER protein homeostasis. One of the main sources of ROS generation in cells is the electron transport chain in mitochondria. Different types of ROS are capable of disturbing protein folding in ER and inducing ER stress. Different studies have shown that ER and mitochondria are in close association via the mitochondrial‐associated membranes (MAM).^154^, ^155^ Diffusion of ROS produced in mitochondria through these membranes enable them to take part in ER redox homeostasis. Therefore, any stressful condition which results in overexpression of mitochondrial ROS can theoretically induce perturbation in ER redox homeostasis and trigger ER stress, a condition which is characterized by accumulation of unfolded proteins in the ER. In consequence of ER stress, cells begin to initiate UPR to counteract stressful condition. The main consequence of UPR is potentiation of protein folding capacity and decreasing protein folding overload.^156^

During normal condition, the ER stress sensors, namely PERK, ATF6 and IRE1, are bound with Bip/GRP78 chaperones and are in an inactive state. However, accumulated unfolded protein during stressful condition promotes separation of chaperones from these three ER stress sensors and makes them active. Activated IRE1α and PERK become oligomerized and transphosphorylated in their cytosolic effector region. Activated ATF6, however, is transported to the Golgi apparatus, where it becomes cleaved to S1P and S2P. The signalling pathways underlying these sensors in next plate promotes activation of a number of transcription factors including Nrf2, NF‐κ B, CHOP, ATF4 and XBP1 as well as several protein kinases including JNK and AKT which, in turn, promotes cellular stress response consisting of induction of chaperons, proteasome degradation pathway, autophagy, ER expansion and finally enhancement of antioxidant defence capacity. In addition, PERK can phosphorylate elF2α which, in turn, can suppress total mRNA translation in stressed cells.^157^

PERK is the main component of UPR in maintaining redox homeostasis. It has been shown that PERK can act as an upstream activator of Nrf2, the most well‐known transcription factor activating expression of a vast variety of genes encoding antioxidant factors and repressing the pro‐oxidant ones. Activated PERK during stress condition phosphorylates Nrf2 and dissociates from Keap1. Released Nrf2 in next place translocate to the nuclease where it binds with promoter region of genes encompassing antioxidant response element and subsequently induces alterations in gene transcription.^158^, ^159^ Furthermore, Nrf2 and ATF4 can form heterodimers which can bind with stress response element and trigger expression of haem oxidase‐1 gene.^160^ Furthermore, IRE1α can also induce autophagy through modulating JNK pathway. JNK phosphorylates Bcl‐2 which results in dissociation of Beclin1 from Beclin1/Bcl‐2 complex. Released Beclin1 in next place initiates formation of Vps34‐Beclin1 complex which accelerates isolated membrane nucleation and formation of autophagosomes. Also, activated XBP1 by IRE1α RNase domain can also induce autophagy by activating transcription of Beclin1. Finally, PERK pathway can also regulate autophagy by means of activating a number of transcription factors including CHOP, ATF4 and Nrf2.^157^

Different studies have depicted putative effects of ELF‐EMF exposure on induction of ER stress. For instance, Chen et al.^161^ have shown that exposure to a picosecond pulsed electric field not only promote mitochondrial apoptosis pathway, but also increases expression of ER chaperons including Grp78 and Grp94, and CHOP. Further studies by Keczan et al. demonstrated that ELF‐EMF exposure effects on ER stress are also cell type dependent. They reported that exposure to pulsed EMF did not affect ER stress markers in HepG2 liver carcinoma and HEK 293T human embryonic kidney cell lines while similar exposure condition promoted expression of ER stress markers in HeLa human cervical cancer cell line.^162^

### 4.3 Autophagic response

Observed during nutrition starvation or related stressful metabolic condition, autophagy is defined as a catabolic event through which cellular components are degraded and recycled following transportation in to lysosomes via specific bilayer structures named autophagosomes.^163^ It has been shown that other stressful conditions such as hypoxia or oxidative stress are also capable of inducing autophagy. Likewise, several chemotherapy agents including arsenic trioxide and oxaliplatin are capable of inducing autophagy.^164^, ^165^ PI3K type III‐Atg6/Beclin 1 complex is responsible for initiation of autophagosomes nucleation while Atg12‐Atg5 and Atg8/LC3‐phosphatidylethanolamine conjugates monitor the process of autophagosomes elongation. These two processes are considered as the main characteristics of autophagy.^163^, ^166^, ^167^ It is noteworthy to mention that while accumulation of a large body of autophagic vacuoles may result in initiation of autophagic cell death, a controlled autophagic response guarantees physiological recycling of damaged organelles and biomacromolecules to cope with energy demands following exposure to cytotoxic drugs or a stressful condition.^168^, ^169^, ^170^

As mentioned above, ROS accumulation can trigger initiation of autophagy which in turn, can promote clearance of extra cellular ROS and prevent oxidative damage occurrence in cells.^171^, ^172^ Since exposure to ELF‐EMF can alter concentrations of cellular ROS, autophagy may also become activated in response to exposure. With this in mind, Chen et al. exposed mouse embryonic fibroblasts (MEF) to PFMF with an intensity of 2 mT and frequency of 50 Hz for different time periods (0.5, 2, 6, 12 and 24 h) and examined alterations in autophagy biomarkers. Based on their results, at 6 h time point, a statistically significant increase in autophagic biomarkers including LC3‐II and formation of GFP‐LC3 puncta was observed. Furthermore, examining cells at this time point with transmission electron microscope demonstrated a statistically significant increase in number of autophagic vacuoles. Using chloroquine, they further confirmed that these alterations in markers of autophagy were resulted from enhancement in autophagic flux and did not take place as a consequence of inhibition of lysosomal function. Investigating the molecular pathway underlying PFMF‐induced autophagy, they observe that the process was totally independent from mammalian target of rapamycin (mTOR) signalling pathway and was largely mediated by cellular ROS.^173^

### 4.4 NF‐kB inflammatory response

Based on different studies, during oxidative stress, activation of NF‐κB transcription factor can protect cells from injury through inhibition of ROS accumulation. Suppressing activation of NF‐κB has shown to be together with an enhancement in TNFα‐induced ROS generation, oxidation of proteins and peroxidation of lipids.^174^ In addition, NF‐κB can also modulate activation of autophagy which is another efficient protective mechanism against oxidative stress. Exposing retinal pigment epithelial cells to different concentrations of H_2_O_2_, a potent inducer of oxidative stress, resulted in phosphorylation of p65 subunit of NF‐κB which, in turn, promoted upregulation of p62 which is a potent promoter of autophagy.^175^ ROS can also delay inactivation of JNK pathway by inhibiting phosphatases responsible for inactivating JNKs. This is mainly mediated through conversion of the catalytic cysteine of these enzymes to sulfenic acid.^176^ Studies have shown an increase in TNF‐alpha‐mediated apoptosis in response to a decline in NF‐κB‐mediated inhibitory effect on JNK activation.^177^ Therefore, it has been proposed that the anti‐apoptotic effect of NF‐κB may be partly mediated through suppression of JNK pathway activation, in which ROS maybe the bridging molecule.^178^ Reported by Wu et al.,^179^ suppressing activation of NF‐κB during recovery period from a temporarily induced oxidative stress condition resulted in a statistically significant decline in cell viability which further confirms the vital role of NF‐κB activation in cell recovery.

Activation of NF‐κB has also been linked to upregulation of expression of several antioxidant targets. One of these prominent targets is manganese superoxide dismutase (MnSOD).^180^ NF‐κB activation following treatment of Ewing's sarcoma cells with TNF‐α resulted in a statistically significant increase in amounts of both thioredoxin and MnSOD.^174^ In addition, ferritin heavy chain is another prominent antioxidant which is significantly upregulated by NF‐κB after treatment with TNF‐α. This protein is mainly responsible for suppressing reaction of iron with H_2_O_2_ and subsequently, inhibiting formation of highly reactive hydroxyl radicals.^181^ Other antioxidant targets affected by NF‐κB consist of glutathione S‐transferase, NAD(P)H dehydrogenase, metallothionein‐3 and glutathione peroxidase‐1.^178^

Examining putative role of ELF‐EMF on NF‐κB, Kim et al.^182^ demonstrated that exposing RAW264.7 cells results in enhanced translocation of phosphorylated NF‐κB in to the nucleus and induction of inflammatory responses. Contrarily, however, Vianale et al.^183^ reported a statistically significant decrease in RANTES, MCP‐1, MIP‐1α and IL‐8 production following ELF‐EMF exposure in HaCaT human keratinocyte cells which was attributed to the inhibition of NF‐κB pathway. These controversial results may be in part due to the different nature of cells used in these studies.

## 5 ANTI‐/PRO‐APOPTOTIC EFFECTS OF ELF‐EMF: STATEMENT OF THE CONTROVERSY

As discussed above, a vast variety of densities ranging from a few micro Tesla up to tens of milli tesla have been applied in studies examining effects of ELF‐EMF on apoptosis.^184^, ^185^, ^186^ In addition, in some cases, promotion of apoptosis by ELF‐EMF exposure was examined in the presence of a co‐stressor (eg chemotherapeutic agents). One obvious result of these studies is that contrary to chemotherapeutic agents, ELF‐EMF exposure does not demonstrate a clear dose–response pattern. In another words, increase in intensity of magnetic fields does not necessarily result in enhancement of apoptosis or other biological effects. Also, no threshold can be considered for induction of ELF‐EMF biological effects. Despite this, another conclusion from reported data is that very low magnetic flux densities and exposure time are also enough for induction of biological responses. Based on these facts, the differential responses to magnetic fields cannot be attributed to the exposure conditions. Instead, biological state of the experiment including the studied cell type or animal tissue, time point after exposure which the assays are performed, density of cells seeded in plate, or other experimental conditions of the study may determine whether ELF‐EMF exposure can induce biological effects. Interestingly, all these factors can significantly affect the extension of ROS produced in response to exposure to ELF‐EMF. Trying to explain these controversies, McCreary et al.^187^ studied fluctuations in cytosolic Ca^2+^ concentrations in Jurkat E6.1 cells following exposure to ELF‐EMF and stated that magnetic fields effects on Ca^2+^ can be only consistently detected when biological characteristics of system including pH of the environment, cell cycle phase and response to a Ca^2+^ agonist were specifically determined. Likewise, several other investigators have also listed some specific criteria necessary to be fulfilled in order to record a consistent response to ELF‐EMF exposure.^188^, ^189^, ^190^ In situations where a co‐stressor such as treatment with a chemotherapeutic agent is also exist, prediction of results becomes more complex. One of the main points, which must be considered additionally in this context, is that how the study has been scheduled. In another words, how is the order of treating with ELF‐EMF exposure and the co‐stressor. In order to make discussion easier, in this section, we only focus on the controversies in apoptosis induced by ELF‐EMF exposure at the presence of another stressor and will not consider the discrepancies related to the biological nature of the materials in the study. In next few paragraphs, we will classify studies based on their experimental design, propose our theory and enumerate the cues in support of the study.

Based on the experimental design of studies existing in literature, we broadly classified treatments into three subclasses: the first group consisted of studies where ELF‐EMF exposure was performed prior to treatment with chemotherapeutic agent. Second group encompassed studies where ELF‐EMF and chemotherapy were performed simultaneously, and finally, the third group consisted of studies where ELF‐EMF exposure was performed after treatment with chemotherapeutic agent. Data regarding the characteristics of ELF‐EMF, duration of exposure, cell type and chemotherapeutic agents applied in the study have been summarized in Table 2. Interestingly, in majority of cases where ELF‐EMF exposure was performed prior to the administration of chemotherapy regimen, the pro‐apoptotic effects of chemotherapeutic agents were reduced. However, if exposure to ELF‐EMF was performed either simultaneously or after pretreatment with chemotherapeutic agent, the pro‐apoptotic effect of regimen was significantly increased. Few exceptions from this pattern however exist in second group. In this manner, simultaneous exposure to ELF‐EMF and chemotherapy with puromycin,^10^ camptothecin (only for 24‐h exposure experiment)^191^ and melatonin^15^ protected cancer cells from pro‐apoptotic effects of these agents. Other important finding of these studies was that exposure to ELF‐EMF per se is not enough for induction of apoptosis.

**TABLE 2:** Consequence of different sequential ELF‐EMF/stressor exposure on induction of apoptosis

Classification of studies		ELF‐EMF treatment	Cell line	Agent co‐used (co‐stressor)	Interaction
ELF‐EMF exposure prior to co‐stressor					
Kaszuba‐Zwoinska et al.^10^	Pulsed electromagnetic field	50 Hz, 45 ± 5 mT, 4 h/stimulation, 3 times in 24 h	Monocytic cell line MonoMac6	Minocycline puromycin, colchicine, cyclophosphamide, hydrogen peroxide	Diminished amount of apoptotic and necrotic cells; enhanced expression of gene belonging to pro‐apoptotic family of Bcl‐2 and AIF agent (antagonism)
Harland et al.^210^	Environmental‐level magnetic fields	60 Hz, 1.2 µT 6 days	MCF‐7	Tamoxifen Melatonin	Significantly block the growth inhibitory action (antagonism)
Palumbo et al.^14^	ELF‐EMF	Intermittent 50 Hz, 1 mT; 1 h	Jurkat cells	Anti‐Fas	Significant decrease of anti‐Fas‐induced apoptosis (antagonism)
Mansourian et al.^16^	Static (DC) magnetic fields	93.25–159.4 µT; 10 min	Erythroleukaemia K562	Electrochemotherapy	Can incur resistance of the cells in response to electric pulses (antagonist)
Falone et al.^196^	ELF‐EMF	75 Hz, 1 mT 5–10 days	SH‐SY5Y human neuroblastoma	H_2_O_2_ Doxorubicin	Reduced vulnerability against both H_2_O_2_ and ROS‐generating doxorubicin (antagonism)
De Nicola et al.^13^	ELF‐EMF	100 mT,N/A; 4 h	U937 cells	Puromycin	Protect U937 from apoptosis (antagonist)
Osera et al.^107^	Pulsed EMF	75 Hz, 2 mT 40 min	SH‐SY5Y cell line	H_2_O_2_	Protected SH‐SY5Y cell line (antagonist)
Falone et al.^193^	ELF‐EMF	75 Hz, 2 mT	SK‐N‐BE(2) neuroblastoma	H_2_O_2_	Reduced vulnerability against H_2_O_2_ (antagonist)
Simultaneous exposure to ELF‐EMF and co‐stressor					
Marcantonio et al.^211^	ELF‐EMF	50 Hz, 1 mT 24–72 h	Neuroblastoma BE(2)C	All trans retinoic acid (ATRA)	Decreased cellular proliferation and increased proportion of G0/G1 phase cells (potentiation)
Kaszuba‐Zwoinska et al.^10^	Pulsed EMF	50 Hz, 45 ± 5 mT 12 h	Neuroblastoma (U937)	Puromycin Cyclophosphamide H_2_O_2_ Colchicine	PEMF protects U937 cells against puromycin‐induced cell death (antagonism)
Baharara et al.^10^	ELF‐EMF	50 Hz, 20 mT 2 h	A2780 ovarian cancer cells	Cisplatin	Increased apoptotic as well as necrotic cells (potentiation)
Ding et al.^8^	ELF‐EMF	60 Hz, 5 mT 24 h	Leukaemia HL‐60	H_2_O_2_	Changes generated by the ELF‐EMF can make resistant cells sensitive (potentiation)
Liburdy et al.^191^	DC Fields	12–50 Hz, 6.5 mT	MCF‐7	Melatonin	Increased the number of apoptotic and necrotic cells (potentiation)
Cid et al.^15^	ELF‐EMF	50 Hz, 10 µT 90 h	HepG2	Melatonin	Enhancement of proliferation by blocking melatonin's oncostatic action (antagonism)
Pirozzoli et al.^12^	ELF‐EMF	50 Hz, 1 mT 3 days	Neuroblastoma cell line LAN‐5	Camptothecin	Enhancement of proliferation by blocking melatonin's oncostatic action (antagonism)
Brisdelli et al.^17^	ELF‐EMF	50 Hz, 1 mT 72 h	K562 cells	Quercetin	Protective effect towards apoptosis only at 24 h exposure (antagonism)
ELF‐EMF exposure following co‐stressor					
Jian et al.^9^	Intermittent	100 Hz, 0.7 mT 1–3 h	BEL‐7402	X‐ray radiotherapy	Significantly higher apoptosis rates (potentiation)
Ruiz‐Gomez et al.^212^	Pulsed EMF	1–25 Hz, 1.5 mT; 1 h	Human colon adenocarcinoma (HCA)	Vincristine Mitomycin Cisplatin	Increased cytotoxicity (potentiation)

### 5.1 Theory: Cellular stress response to ELF‐EMF protects cells from chemotherapy‐induced apoptosis

**FIGURE 3:**
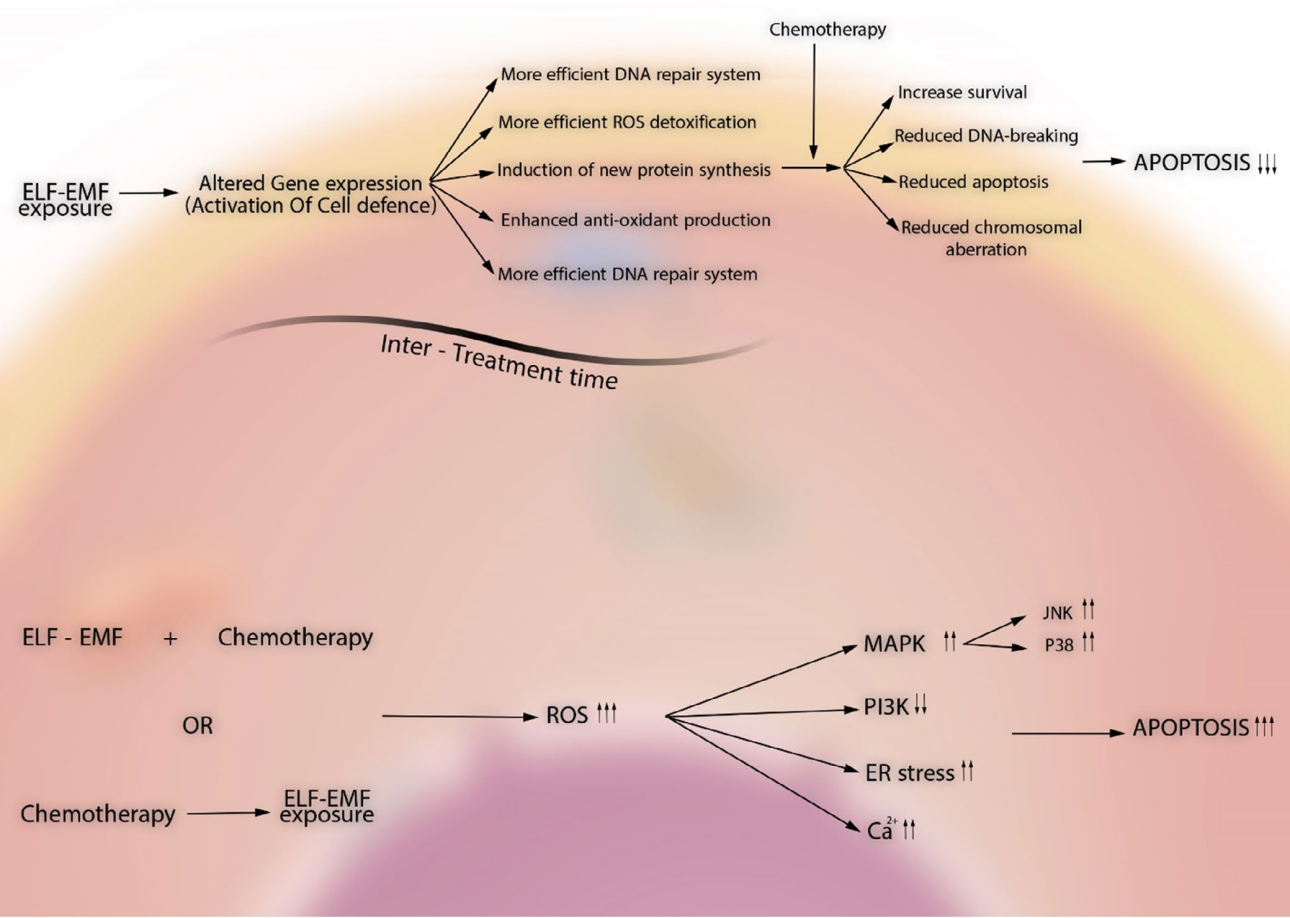
A schematic illustration of the hypothesis for explanation of controversial effects of ELF‐EMF on apoptosis. Upper side: ELF‐EMF exposure prior to treatment with the apoptosis‐inducing agent will result in activation of cellular defence system and alteration in expression of a number of genes which, in next place, will end in promotion of DNA repair system, ROS detoxification system and Ca^2+^ homeostasis through production of new protective proteins and antioxidative enzymes or restoration of antioxidative stress molecule reservoirs such as glutathione and so on. In next place, upon introduction of the apoptosis‐inducing agent, cells will defend themselves with robust protective system and consequently, lower rate of apoptosis will occur. Lower side: Contrarily, ELF‐EMF co‐treatment with or immediately after chemotherapeutic agent will enhance the rate of injury by ROS overproduction or unbalancing Ca^2+^ homeostasis which will end in promotion of apoptosis

As mentioned in previous sections, studies in the literature have demonstrated that ELF‐EMF exposure can effectively activate adaptive response and underlying pathways in cells without significantly affecting cellular viability. Consistently, numerous studies have shown that cells are physiologically well buffered against negative effects of ELF‐EMF in monotherapies. Nevertheless, upon addition of even a weak co‐stimulator, (eg upon exposure to a toxin, viruses, or a DNA‐alkylating agents, as well as stressful environmental conditions including a hypoxic incubation area, hyperthermic microenvironment, or under oxidative stressful condition), tolerable ELF‐EMF exposure will meaningfully increase the number of apoptotic cell deaths.^101^ Finally, sequential pretreatment with ELF‐EMF and then exposure to apoptotic agents could meaningfully increase the tolerability of the cells to anti‐neoplastic agents and reduce cell deaths (Figure 3). Clearly, considering the sequential events in this scenario, ELF‐EMF pretreatment had induced a series of protective events which could have an antagonizing effect on pro‐apoptotic effects of the anti‐neoplastic agent. Since the only proven effects of ELF‐EMF exposure on cells are cellular adaptive responses, ROS overproduction and intracellular calcium overload, from which the first one is only protective and the other two can be significantly deleterious upon co‐treatment and pretreatment, we hypothesized that the nature of studies (ie the relation between the time of ELF‐EMF exposure and treatment with pro‐apoptotic agent) may be the result beneath this controversy.

Numerous data exist in support of this theory. For instance, when SH‐SY5Y neuroblastoma cells were concurrently exposed to H_2_O_2_ and ELF‐EMF with intensity of 1 mT and frequency of 50 Hz for 24 h, the increase in catalase was significantly restricted.^192^ Contrarily, reports have shown that exposure to magnetic fields with above‐mentioned characteristics alone can induce expression of cytochrome P450 (CYP450) and glutathione S‐transferase (GST), both of which play key roles in cellular detoxification process.^192^, ^193^ Reported by Patruno et al.,^194^ administration of phorbol 12‐myristate 13‐acetate (PMA) to human erythro‐leukaemic cells following exposure to ELF‐EMF with intensity of 1 mT and frequency of 50 Hz more effectively enhanced the activity of CYP450. Recently, it has been shown that long‐term exposure of SH‐SY5Y cells to 50 Hz, 1 mT ELF‐MF significantly enhances CAT and GPX free radical scavenging activity and improves reduced glutathione's availability.^195^, ^196^ This is important, as GSH is a vital co‐factor for GPX and several other enzymes involved in phase II drug metabolization.^197^, ^198^, ^199^ Recently, it has been shown that long‐term exposure to ELF‐EMF before exogenous treatment with methylglyoxal (MG) significantly reduces susceptibility of cancer cells to cytotoxic effects of this agent.^195^ This has been mainly attributed to the enhanced accessibility of GSH following ELF‐EMF exposure which is an important co‐factor for directing MG into the glyoxalase‐mediated detoxifying system.^200^ Recently, it has also been shown that ELF‐EMF is capable of inducing sirtuin 3 (SIRT3) expression.^196^ The signalling cascade mediated by SIRT3 is capable of improving mitochondrial integrity and fitness following exposure to oxidative proteotoxic stress.^201^ Finally, activation of Nrf‐2 following exposure to 50 Hz, 1 mT ELF‐MF has shown to be in association with development of resistance to ROS‐producing chemotherapeutic agents in different types of cancer cells.^202^, ^203^, ^204^, ^205^

### 5.2 Possible explanation for exceptional results

As stated above, few exceptions from this pattern however exist in second group. In this manner, simultaneous exposure to ELF‐EMF and chemotherapy with puromycin, camptothecin (only for 24‐h exposure experiment) and melatonin, protected cancer cells from pro‐apoptotic effects of these agents. These observations, however, can be easily explained based on the nature of the anticancer agent applied. For instance, melatonin is a potent free radical scavenger and several points of evidence exist that it can balance upregulated ROS content of cells. In cancer cells, upregulated ROS levels result in activation of tyrosine kinase pathway inhibitors including transcription factor and promote cellular growth. Administration of melatonin in cancer cells modulates ROS levels, inhibits NF‐kB activation and suppresses tumour growth.^206^ Contrarily, when cells are exposed to ELF‐EMF, a new source of ROS production is introduced in cells which can at least partially reverse anticancer effects observed with cell's treatment with melatonin.

Camptothecin is a unique chemotherapeutic agent which induces apoptosis at S phase of cell cycle through inhibition of topoisomerase I.^207^ Thus, depending on the doubling time of the cells, a specific time period is required for initiation of anticancer effects of camptothecin which is usually about 24 h. Thus, when cells are simultaneously exposed to ELF‐EMF and camptothecin, during the first 24 h, cells have enough time to undergo cellular stress response prior to initiation of action of camptothecin, and therefore, it is not surprising to observe protective effects against apoptosis. However, when the exposure time extends to 48 h, camptothecin is completely active and have produced excess ROS levels through which induced cellular stress response is not capable of coping with it, and therefore, extra ROS produced by ELF‐EMF can help in promotion of apoptosis.

Puromycin is a specific chemotherapeutic agent which is capable of inducing cell death through inhibition protein synthesis, accelerating accumulation of misfolded proteins and induction of apoptosis.^208^ Grassi et al.^209^ have shown that pre‐exposing cells to ELF‐EMF can significantly reduce apoptosis induced by puromycin which is consistent with the cellular stress response theory. However, the study by Kaszuba‐zwoinska et al.^10^ also showed that simultaneous treatment with ELF‐EMF and puromycin can also protect from apoptotic effect. As protein synthesis process and accumulation of unfolded proteins in cells also requires a time period which is usually about 10–12 h, it can also be concluded that cells during concurrent treatment also have enough time for adaption. It is also noteworthy to mention that observed protective effect in this study was very low and about 5%–10% in its highest point.^10^

## 6 CONCLUSION AND FUTURE PERSPECTIVE

As discussed herein, cellular stress response is a unique behaviour of cells following exposure to ELF‐EMF which helps them to cope with next more stressful encountered conditions. This response is mainly due to the mild increase in cellular ROS levels mediated by ELF‐EMF exposure which is not capable of inducing apoptosis alone. The statement discussed in present article, if proved to be true, will become very important in designation of new therapeutic schedules for treatment of cancer as concurrent ELF‐EMF exposure/chemotherapy or ELF‐EMF exposure immediately following chemotherapy can significantly improve pro‐apoptotic effects of chemotherapeutic agents. Furthermore, following this hypothesis, one can apply ELF‐EMF in treatment of resistant cancers as one of the main mechanisms of resistance to chemotherapeutic agents is high capacity of these cells in scavenging free radicals. Additive effects of ELF‐EMF to chemotherapeutic agents in induction of an oxidative stress condition may be helpful in this context. More importantly, ELF‐EMF exposure to normal cells in most cases has shown to be safe and un‐harmful. Therefore, ELF‐EMF therapy may not pose any other adverse effects except for those observed with potentiation of chemotherapeutic agents’ cytotoxic effects. As discussed herein, determination of ELF‐EMF’s intensity window is also very important as no response occurs outside this range, making it a highly personalized therapy. Finally, although this hypothesis apparently sounds rational, future studies comparing results of ELF‐EMF exposure before, during and immediately after chemotherapy is highly recommended for further confirmation of this theory.

## CONFLICT OF INTEREST

Authors declare that they have no conflict of interest.

## AUTHORS’ CONTRIBUTION

MB, MAJ and AM drafted the main body of the manuscript; BD and MRE modified the manuscript and extended some sections and also designed illustrations; SPS and AMA conceived the original idea of the manuscript; and AMA furthermore supervised the team.

## Data Availability

The data that support the findings of this study are available from the corresponding author upon reasonable request.
